# Development of MALDI-TOF mass spectrometry for the identification of lice isolated from farm animals

**DOI:** 10.1051/parasite/2020026

**Published:** 2020-04-30

**Authors:** Basma Ouarti, Maureen Laroche, Souad Righi, Mohamed Nadir Meguini, Ahmed Benakhla, Didier Raoult, Philippe Parola

**Affiliations:** 1 Aix Marseille Univ., IRD, AP-HM, SSA, VITROME 13005 Marseille France; 2 IHU-Méditerranée Infection 19–21 Boulevard Jean Moulin 13005 Marseille France; 3 Université Chadli Bendjdid, Département des sciences Vétérinaire 36000 El Tarf Algeria; 4 Institut des Sciences Vétérinaire et Agronomiques, Université Mohamed Cherif Messaadia 41000 Souk-Ahras Algeria; 5 Aix Marseille Univ., IRD, AP-HM, MEPHI 13005 Marseille France

**Keywords:** MALDI-TOF MS, Lice, Phthiraptera, Anoplura, Mallophaga

## Abstract

Matrix-assisted laser desorption/ionization time-of-flight mass spectrometry (MALDI-TOF MS) is now routinely used for the rapid identification of microorganisms isolated from clinical samples and has been recently successfully applied to the identification of arthropods. In the present study, this proteomics tool was used to identify lice collected from livestock and poultry in Algeria. The MALDI-TOF MS spectra of 408 adult specimens were measured for 14 species, including *Bovicola bovis, B. ovis, B. caprae, Haematopinus eurysternus, Linognathus africanus, L. vituli, Solenopotes capillatus*, *Menacanthus stramineus*, *Menopon gallinae, Chelopistes meleagridis*, *Goniocotes gallinae*, *Goniodes gigas, Lipeurus caponis* and laboratory reared *Pediculus humanus corporis*. Good quality spectra were obtained for 305 samples. Spectral analysis revealed intra-species reproducibility and inter-species specificity that were consistent with the morphological classification. A blind test of 248 specimens was performed against the in-lab database upgraded with new spectra and validated using molecular tools. With identification percentages ranging from 76% to 100% alongside high identification scores (mean = 2.115), this study proposes MALDI-TOF MS as an effective tool for discriminating lice species.

## Abstract (fr)

La Spectrométrie de Masse à Temps de Vol par Désorption/Ionisation Laser Assistée après Matrice est maintenant utilisée pour l’identification rapide des microorganismes isolés à partir d’échantillons cliniques et a récemment été appliquée avec succès pour l’identification des arthropodes. Dans cette étude, cet outil protéomique a été utilisé pour identifier les poux prélevés sur le bétail et la volaille en Algérie. Les spectres MALDI-TOF MS de 408 spécimens adultes ont été mesurés pour 14 espèces, dont *Bovicola bovis*, *B. ovis*, *B. caprae*, *Haematopinus eurysternus, Linognathus africanus*, *L. vituli*, *Solenopotes capillatus*, *Menacanthus stramineus*, *Menopon gallinae*, *Chelopistes meleagridis*, *Goniocotes gallinae*, *Goniodes gigas*, *Lipeurus caponis* et *Pediculus humanus corporis* élevé en laboratoire. Des spectres de bonne qualité ont été obtenus pour 305 échantillons. L’analyse spectrale a révélé une reproductibilité intra-espèce et une spécificité inter-espèces qui concordaient avec la classification morphologique. Un test à l’aveugle de 248 échantillons a été effectué par rapport à la base de données de notre laboratoire mise à niveau avec de nouveaux spectres et validée à l’aide d’outils moléculaires. Avec des pourcentages d’identification allant de 76 à 100 % et des scores d’identification élevés (moyenne : 2,115), cette étude propose MALDI-TOF MS comme un outil efficace pour distinguer les espèces de poux.

## Introduction

Lice are highly host-specific insects [20], belonging to the order Phthiraptera. They are obligate parasites of birds and many species of mammals, including humans [40, 41]. Nearly 5000 species of parasitic lice have been described and classified under four sub-orders: Anoplura, Amblycera, Ischnocera, and Rhynchophthirina. Anoplura (sucking lice) are hematophagous and feed exclusively on mammals [34]. The lice of the other suborders are Mallophaga (chewing lice). These lice mostly infest birds, and secondarily mammals, and feed on feathers, dead skin, blood or secretions from their hosts [18].

Lice parasitism may be responsible for pediculosis causing mild to severe anemia, and many types of skin damage such as focal necrosis and scars on the skin of heavily infested animals [8, 12]. These have economic consequences especially for livestock farmers [8, 45]. Some sucking lice such as *P. humanus corporis* (*Pediculus humanus corporis*) have the ability to transmit pathogens to humans [17].

The identification of arthropods including lice is an important step for surveillance and control of parasitism as well as transmitted diseases [25]. Currently, lice are mainly identified morphologically based on dichotomous keys that take high consideration of the host animal from which the louse has been collected [32, 47].

Morphological identification requires entomological expertise and specific documentation [50]. For lice and other arthropods, it may be limited by the integrity of the specimen which can be damaged during collection or transport by its fragility or by the absence of distinctive morphological criteria at an immature stage of the life cycle such as ticks [33].

Alternative methods such as molecular approaches have been developed to identify arthropods including lice [19, 27]. These are based on comparative analyses of gene sequences such as the *18S* rRNA or the cytochrome c oxidase subunit I (*COI*) genes widely used for the identification of lice [19, 27]. However, the NCBI GenBank database is still far from comprehensive regarding animal lice gene sequences [24].

Matrix-assisted laser desorption/ionization time-of-flight mass spectrometry (MALDI-TOF MS) is an ionization technique that generates specific spectra from protein extracts from organisms [48]. The acquisition of the spectra allows the creation of a database based on reference spectra of the formally identified organism [25]. In recent years, this proteomic approach has revolutionized clinical microbiology for the identification of bacteria and fungi [36, 39].

Recently, MALDI-TOF MS has been evaluated as an efficient tool for the identification of arthropods including ticks [2, 5], mosquitoes [26, 28, 46], culicoides [6, 35], fleas [30, 48], triatomines [25], tsetse flies [15], and phlebotomines [9] in laboratory and field conditions.

The objective of the present study was to test the ability of MALDI-TOF MS to identify lice specimens collected from livestock and poultry in Algeria.

## Materials and methods

### Ethical considerations

Informal verbal consent was obtained from the owners of the mammals and poultry that were selected for sampling lice directly. Lice were not sampled from protected animals nor from animals in private residences or national parks.

Human lice were reared at IHU Méditerranée Infection on adult female New Zealand white rabbits obtained from Charles River Laboratories. They were handled according to Decree No. 2013–118, 7 February 2013 and as described in the approved experimental protocols (references APAFIS #01077.02 & 2015050417122619). Protocols were approved by the Ethics Committee “C2EA-14” of Aix-Marseille University, France and the French Ministry of National Education, Higher Education and Research.

### Field capture and morphological identification of lice

The collection was carried out on mammals and poultry between 2015 and 2017 in three regions of northeastern Algeria: El Tarf (36°46′1.2″ N, 8°19′1.2″ E), Souk Ahras (36°17′11″ N, 7°57′4″ E); and Guelma (36°27′0″ N, 7°25′0″ E) during three seasons (autumn, winter, spring).

**Figure 1:**
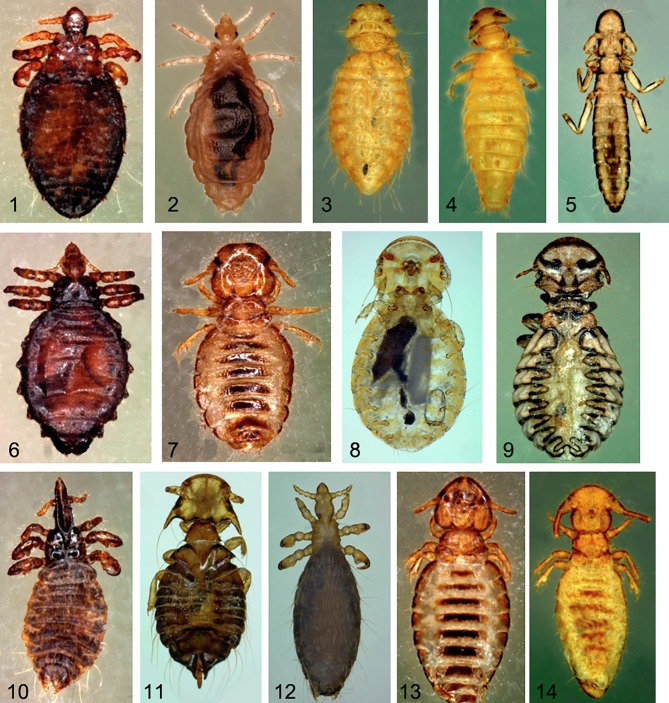
Photographs of habitus of 13 species of lice collected from three regions of northeastern Algeria: *Solenopotes capillatus* (1); *Menopon gallinae* (3); *Menacanthus stramineus* (4); *Lipeurus caponis* (5); *Haematopinus eurysternus* (6); *Bovicola caprae* (7); *Goniocotes gallinae* (8); *Goniodes gigas* (9); *Linognathus vituli* (10); *Chelopistes meleagridis* (11); *Linognathus africanus* (12); *Bovicola bovis* (13); *Bovicola ovis* (14). Laboratory specimens of *Pediculus humanus corporis* (2) were also used in this study.

For mammals, the animals were examined by parting their wool from sheep and goats and hair from cattle, visually inspecting the skin for lice. In poultry, the head and feathers on the neck, feet, skin, wing feathers, feathers of the belly, and feathers of the croup and of the tail were meticulously examined. In some cases, chickens were sprayed with insecticide and placed on a small spot on a sampling surface for 20 min [4]. Lice collected from the same animal were recovered and stored in the same tube either dry at −20 °C or in 70% ethanol to be transported to Marseille, France for further analyses. For the present study, we used frozen lice only and kept the other lice for future studies. Each louse was rinsed with ethanol (70%) for 15 min, and later in distilled water for one minute. All body parts of the collected body lice were examined using a Zeiss Axio Zoom V16 (Zeiss, Marly-le-Roi, France) microscope. The morphological keys provided by Wall [47] and Pajot [32] were used for morphological identification (Fig. 1). The names of the species of lice and their abbreviations used in this study were chosen according to previously published identification keys [1, 7, 14, 34, 37].

### Molecular identification of lice

Following morphological identification, between 8 and 10 specimens of each louse species were selected from at least two animal hosts at each study site. The abdomen of each louse was used for the extraction of DNA using an EZ1 DNA tissue extraction kit (Qiagen, Hilden, Germany), according to the manufacturer’s instructions. Lice DNA was then eluted in 100 μL of Tris EDTA buffer using a DNA extracting EZ1 Advanced XL Robot (Qiagen), as previously described [5]. The DNA was either immediately used or stored at −20 °C until molecular analysis. The DNA extracting EZI (Qiagen) was disinfected after each batch of extraction as per the manufacturer’s recommendations in order to avoid cross-contamination.

SAIDG (5′ – TCTGGTTGATCCTGCCAGTA – 3′) and SBIDG (5′ – ATTCCGATTGCAGAGCCTCG – 3′) primers were used to amplify partial 539 base pair *18S* rRNA gene sequences for species-level molecular identification of the lice, as previously described [21]. The DNA samples tested were successfully amplified using an automated DNA thermal cycler (Applied Biosystems, Foster City, CA, USA). The cycling program consisted of 15 min at 95 °C followed by 39 cycles of denaturing at 95 °C for 30 s, annealing at 58 °C for 30 s, extension of 1 min at 72 °C, followed by a final cycle of 5 min at 72 °C and sampling while held at 4 °C. A mix without DNA was used as a negative control. The amplification products were then subjected to electrophoresis through a 1.5% agarose gel stained with SYBR Safe™ and visualized with the ChemiDoc™ MP ultraviolet imager (Bio-Rad, Marnes-la-Coquette, France).

The positive samples were purified, sequenced using a Big Dye Terminator kit and an ABI PRISM 3130 Genetic Analyzer (Applied BioSystems, Courtaboeuf, France). The obtained sequences were analyzed and assembled using ChromasPro, version 1.34 (Technelysium Pty, Ltd., Tewantin, QLD, Australia).

BioEdit (http://www.mbio.ncsu.edu/BioEdit/bioedit.html) was used for sequence alignment.

All sequences were compared to the GenBank database using BLAST analysis and new sequences were deposited in GenBank (Table 3).

### Sample preparation for MALDI-TOF MS analysis

Two protocols were tested to assess which body part was relevant for MALDI-TOF MS analyses. In protocol 1, the louse was longitudinally cut into two equal parts, one used for MALDI-TOF MS analysis and the other for molecular biology. In protocol 2, a transverse section was performed to separate the cephalothorax and the legs for the MALDI-TOF MS and spectra obtained were tested. We used the abdomen for molecular biology.

Body halves (protocol 1) or cephalothorax-legs (protocol 2) of the selected specimens were individually homogenized using a TissueLyser II device (Qiagen, Hilden, Germany) with three 60-second cycles at a frequency of 30 Hz, in 15 μL of 50% (v/v) acetonitrile (Fluka, Buchs, Switzerland) and 15 μL of 70% (v/v) formic acid (Sigma, Lyon, France) with glass powder (Glass beads, acid-washed G4649, ≤106 μm, Sigma, Lyon. France) in 1.5 mL micro-tubes.

**Figure 2:**
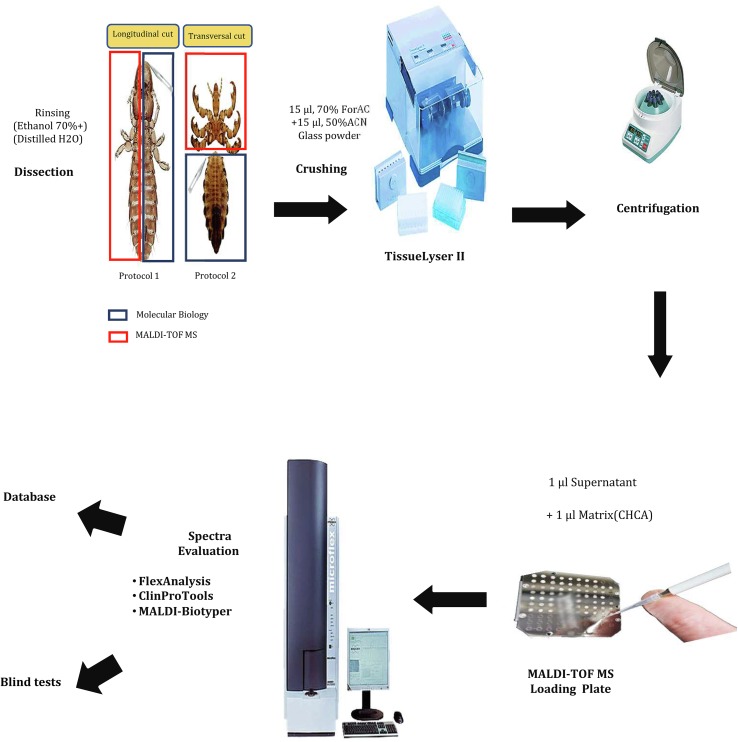
An explanatory flowchart of the MALDI-TOF MS protocol. ACN: Acetonitrile, ForAC: Formic acid, CHCA: α-cyano-4-hydroxycinnamic acid.

In both protocols, after homogenization of the sample, a quick spin centrifugation at 10,000 rpm for 1 min was performed to pellet debris and 1 μL of supernatant from each sample was deposited on the MALDI-TOF MS target plate in quadruplicate (Bruker Daltonics, Wissembourg, France) and covered with 1 μL of CHCA matrix solution composed of saturated α-cyano-4-hydroxycinnamic acid (Sigma), 50% acetonitrile (v/v), 2.5% trifluoroacetic acid (v/v) (Aldrich, Dorset, UK) and high-performance liquid chromatography (HPLC)-grade water. After drying for several minutes at room temperature, the target was placed in the MALDI-TOF MS [30] (Fig. 2).

**Figure 3:**
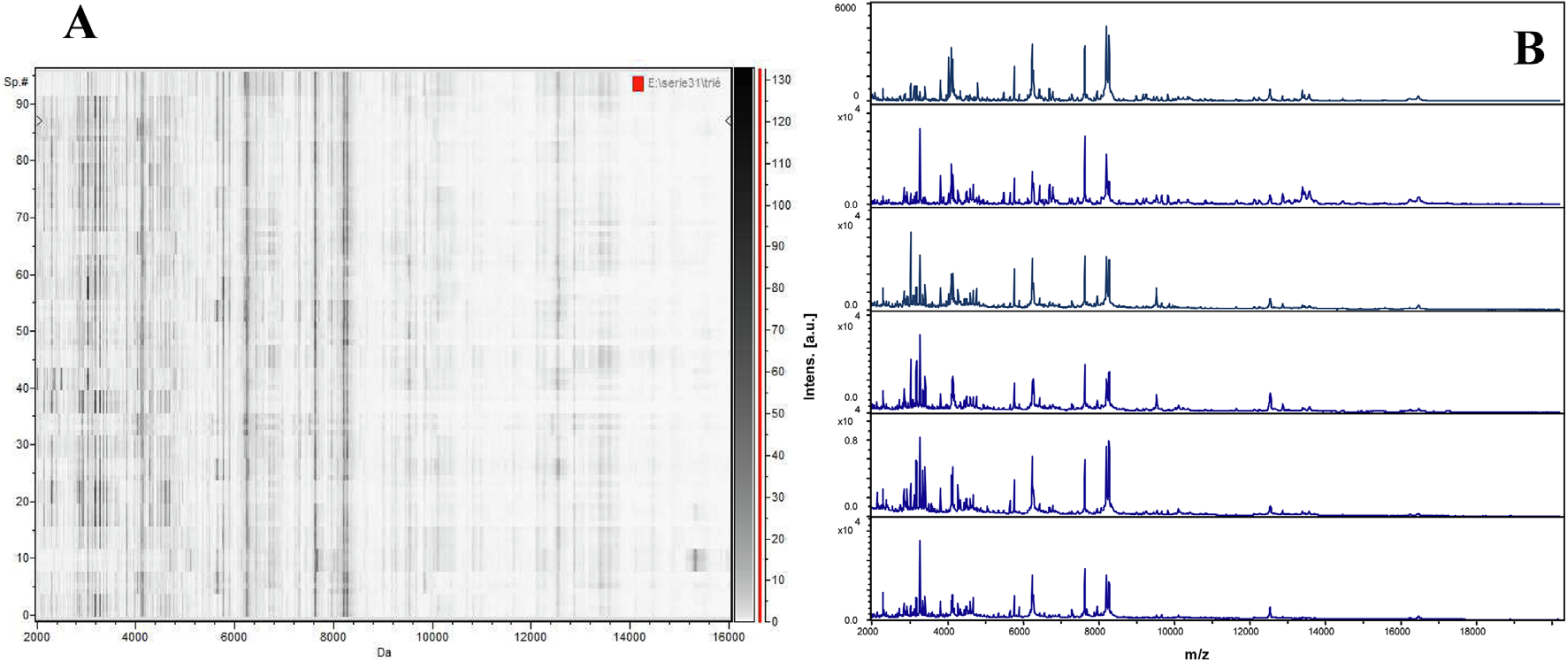
MALDI-TOF analysis of *Pediculus humanus corporis*. (A) MS profiles of 24 specimens using the gel view tool of ClinProTools software, (B) MS profiles of different specimens of *Pediculus humanus* corporis using FlexAnalysis software.

Following comparison of the spectra quality obtained when using protocol 1 and protocol 2, protocol 2 (using cephalothorax-legs) was chosen for further analyses. The validity of the spectra obtained with protocol 2 was confirmed by testing 24 fresh lice *P. humanus corporis* from laboratory rearing by MALDI-TOF MS. Reproducibility and spectra quality was confirmed using FlexAnalysis v.3.3 software and the gel view tool of ClinProTools 2.2 software (Bruker Daltonics, Leipzig, Germany) (Fig. 3). Non-engorged fresh *P. humanus corporis* lice were later used as controls for each MALDI-TOF MS assay.

### MALDI-TOF MS parameters

Protein mass profiles were obtained using a Microflex LT MALDI-TOF Mass Spectrometer (Bruker Daltonics), using Flex Control software (Bruker Daltonics), with the parameters described previously [49]. The profiles of the spectra obtained were viewed using FlexAnalysis v.3.3 software and exported to ClinProTools v.2.2 and MALDI-Biotyper v.3.0 software (Bruker Daltonics) for data processing (smoothing, basic subtraction and peak selection) and cluster analysis.

### Creation of a reference spectra database

In order to obtain reference spectra and upgrade our arthropod database, a subgroup of lice specimens identified both morphologically and using molecular tools were subjected to MALDI-TOF MS (Tables 2 and 3). Lice species from the same genus were run on the same MALDI-TOF MS target plate to rule out any plate bias. Intra-species reproducibility and inter-species specificity of MALDI-TOF MS spectra were visually evaluated using the gel view, dendrogram and principal component analysis tools of ClinProTools 2.2 and MALDI-Biotyper v3.0. (Bruker Daltonics). Dendrograms are based on the results of Composite Correlation Index (CCI) matrices. CCIs are calculated by dividing spectra into intervals and comparing these intervals across a dataset. The composition of correlations of all intervals provides the CCI which is used as a parameter that defines the distance between spectra. A CCI match value of 1 represents complete correlation, whereas a CCI match value of 0 represents an absence of correlation [25]. Spectral dendrograms were created to assess the profile diversity within each species and high-quality spectra from separate clusters were selected using FlexAnalysis software v.3.3. (Bruker Daltonics) to update the reference spectra database.

Reference spectra were selected based on intensity, overall spectrum quality and intra-species reproducibility. For each reference sample, a main spectrum profile (MSP) was created using the automated function of MALDI-Biotyper software v.3.3. (Bruker Daltonics). Spectra from a spot of lower quality were sometimes removed to obtain a high-quality MSP. MSPs were created on the basis of an algorithm using peak position, intensity and frequency data. Between two and nine new reference spectra per species were added to the lice database in our laboratory [26].

### Blind tests and cluster analysis

New specimens of lice collected at different study sites were tested. Each spectrum obtained by MALDI-TOF MS analysis as described above was subjected to a blind test analysis against the upgraded database. The significance of the identification was determined using the log score values (LSV) given by MALDI-Biotyper software v.3.3. corresponding to a signal intensity level of the mass spectra of the query and reference spectra. The LSV range was from 0 to 3. LSVs allow for good evaluation of reproducibility between a queried spectrum and a reference spectrum, as they result from a thorough comparison of the position of peaks and the intensity between those two spectra (MALDI BioTyper Help, Bruker). In order to visualize MALDI-TOF MS profile similarities and distances, hierarchical clustering of the mass spectra of all tested species was performed using the dendrogram function of MALDI-Biotyper software v.3.3. Although no threshold has been definitively validated for arthropod identification using MALDI-TOF MS, LSVs ≥ 1.8 were considered adequate for relevant identification, as reported in pioneer papers [30, 44]. Percentages of included spectra are reported in Table 4.

## Results

### Lice collection and morphologic identification

A total of 4112 lice were collected from several livestock farm animals and stored at −20 °C: a total of 23 sheep, 20 cattle, 14 goats, and 13 poultry (Table 1).

**Table 1:** Sampling regions, number of parasitized animals, and specimens of lice collected from sheep, cattle, goats, and poultry in Algeria.

Host	Souk-Ahras	Guelma	El Tarf	Total number of specimens collected
Cattle (*n* = 20)	1066	748	/	1814
Sheep (*n* = 23)	256	300	96	747
Goats (*n* = 14)	288	289	229	806
Poultry (*n* = 13)	615	225		840
Total	2225	1562	325	4112

On the basis of morphological criteria, 13 species of lice were morphologically identified including four species of sucking lice and 9 chewing lice (Table 2). Seven species were collected on mammals including four from cattle with *B. bovis* (*Bovicola bovis*) (*n* = 105), *H. eurysternus* (*Haematopinus eurysternus*) (*n* = 679), *L. vituli* (*Linognathus vituli*) (*n* = 45) and *S. capillatus* (*Solenopotes capillatus*) (*n* = 985), two from goats with *B. caprae* (*Bovicola caprae*) (*n* = 448) and *L. africanus* (*Linognathus africanus*) (*n* = 362), and one species from sheep namely *B. ovis* (*Bovicola ovis*) (*n* = 495). Six other lice species were collected from poultry including *G. gallinae* (*Goniocotes gallinae*) (*n* = 275), *Li. caponis* (*Lipeurus caponis)* (*n* = 61), *M. gallinae* (*Menopon gallinae*) (*n* = 414), *Me. stramineus* (*Menacanthus stramineus*) (*n* = 211), *C. meleagridis* (*Chelopistes meleagridis*) (*n* = 20) and *Go. gigas (Goniodes gigas)* (*n* = 12) (Table 2). Genera abbreviations were modified in this study to properly differentiate genera with the same initials. A list of abbreviations is provided.

**Table 2:** Morphological identification of lice collected from Algeria and stored at −20 °C before being tested by MALDI-TOF MS.

Hosts	Morphological ID	Souk-Ahras	Guelma	El Tarf	Total
Cattle (*n* = 20)	*Bovicola bovis* b	49	56	/	105
	*Haematopinus eurysternus* a	401	278	/	679
	*Linognathus vituli* a	26	19	/	45
	*Solenopotes capillatus* a	646	339	/	985
Sheep (*n* = 25)	*Bovicola ovis* b	289	111	95	495
Goats (*n* = 18)	*Bovicola caprae* b	258	105	85	448
	*Linognathus africanus* a	145	111	106	362
Poultry (*n* = 17)	*Menacanthus stramineus* b	46	162	3	211
	*Menopon gallinae* b	199	83	132	414
	*Chelopistes meleagridis* b	20	/	/	20
	*Goniocotes gallinae* b	101	92	82	275
	*Goniodes gigas* b	2	/	10	12
	*Lipeurus caponis* b	30	17	14	61
Total	13 species	2212	1373	527	4112

a Anoplura.

b Mallophaga.

### Molecular identification of lice

Of the 4112 lice morphologically identified, 159 lice specimens preserved at −20 °C and belonging to 13 species were randomly selected to be included in the study.

Randomly selected specimens of each species were subjected to molecular identification targeting the *18S* rRNA gene. A GenBank request revealed that *18S* rRNA gene reference sequences were available for 8 of the 13 lice species. Amongst these eight available sequences on GenBank, four had an average quality and the remaining four were of very poor quality. No sequences were available for five species including *Li. caponis, C. meleagridis, G. gallinae, Go. gigas*, and *Me. stramineus* (Table 3).

**Table 3:** Results of the molecular identification based on the partial *18S* rRNA of lice: BLAST analysis and sequences deposited on the NCBI GenBank database.

Morphological identification	N	Molecular identification by BLAST (accession number)	Identity level with GenBank	Sequences deposited in GenBank *18S* with accession numbers
*Bovicola bovis* b	17	*Bovicola bovis* (JX184911.1)	100%	MH377332.1
*Haematopinus eurysternus* c	11	*Haematopinus tuberculatus* (GU569180.1)	99.21%	MH377326.1
*Linognathus vituli* b	13	*Linognathus vituli* (JX401573.1)	99%	MH377328.1
*Solenopotes capillatus* b	17	*Solenopotes capillatus* (JX184910.1)	100%	MH377327.1
*Bovicola ovis* b	20	*Bovicola ovis* (GU569184.1)	100%	MH377330.1
*Bovicola caprae* c	20	*Bovicola bovis* (JX184911.1)	99.79%	MH377331.1
*Linognathus africanus* c	15	*Linognathus vituli* (JX401573.1)	95.86%	MH377329.1
*Menacanthus stramineus* a	19	*Menacanthus* sp (AF385066.1)	99.79%	MH377333.1
*Menopon gallinae* c	5	Menoponidae sp (JQ309930.1)	100%	MH377334.1
*Chelopistes meleagridis* a	9	*Rhynonirmus* sp*.* (AF385048.1)	98.98%	MH377335.1
*Goniocotes gallinae* a	5	*Goniodes aff. dissimilis/gigas* (AY077767.1)	97.69%	MH469486.1
*Goniodes gigas* a	2	*Goniodes aff. dissimilis/gigas*(AY077767.1)	97.69%	MH469487.1
*Lipeurus caponis* a	6	*Rhynonirmus* sp*.* (AF385048.1)	99.79%	MH469485.1
13 species	159	/	/	/

N: Number of specimens used for molecular biology.

a Species with sequences not available in GenBank.

b Species with correct identification using the *18S* rRNA gene.

c Species with incorrect molecular identification.

For 13 species of lice, the BLAST analysis of *18S* rRNA reference sequences of the lice specimens of the same species demonstrated high identity ranging from 99% to 100%, supporting correct morphological identification (Table 3). The sequences obtained for each species of lice were corrected and blasted to reveal the intra-species similarity of the sequence of the *18S* RNA gene. Sequence alignment using BioEdit software revealed that all sequences from the same species were identical and thirteen *18S* rRNA gene good quality consensus sequences were deposited in the NCBI GenBank database (Table 3).

### MALDI-TOF MS analyses

A total of 427 lice preserved at −20 °C were tested by MALDI-TOF MS using two protocols.

**Figure 4:**
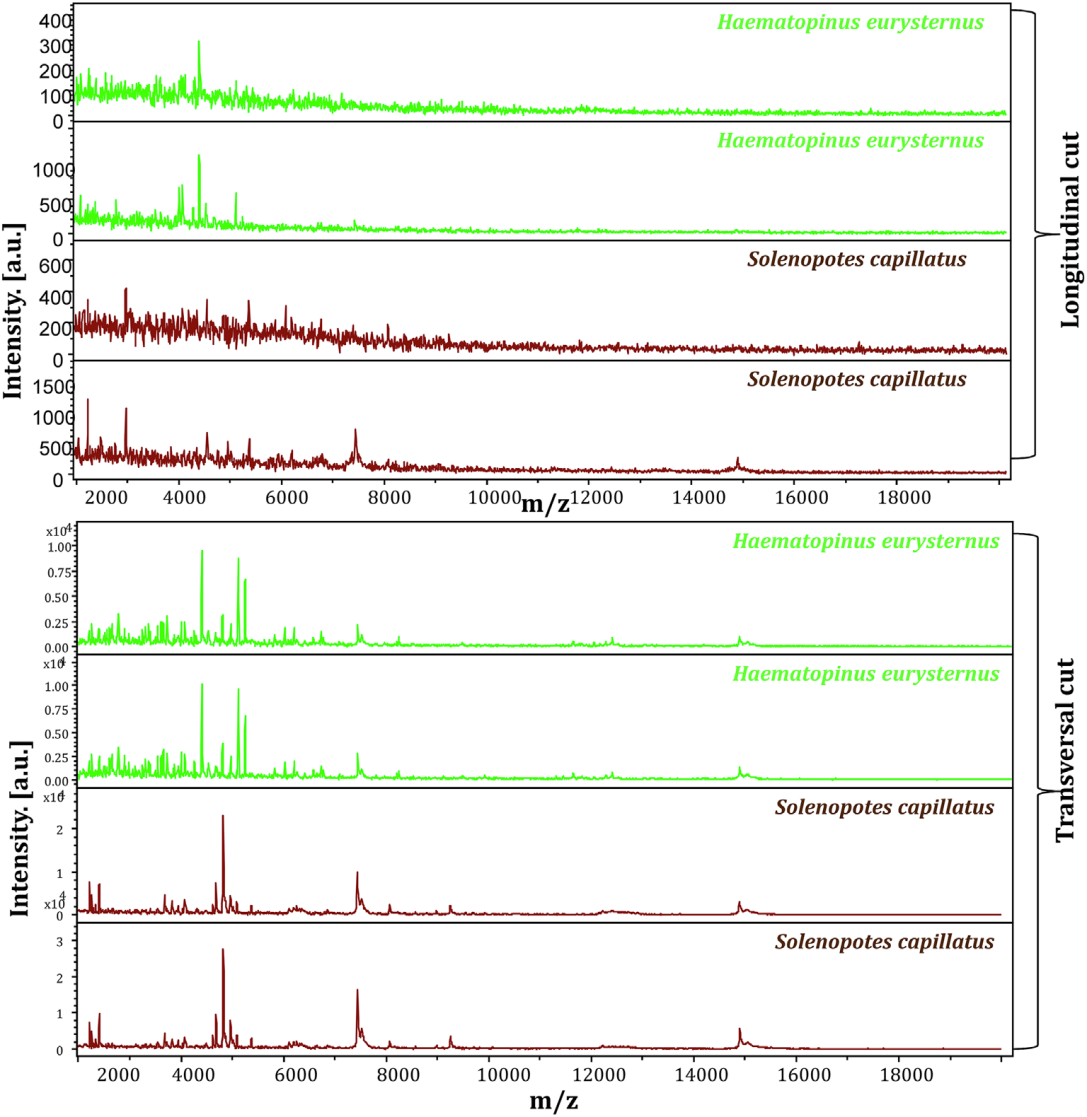
Comparison between the spectra obtained using protein extracts from (A) the cephalothorax and legs; (B) a longitudinal cut of the louse. Spectra were visualized using FlexAnalysis software.

**Figure 5:**
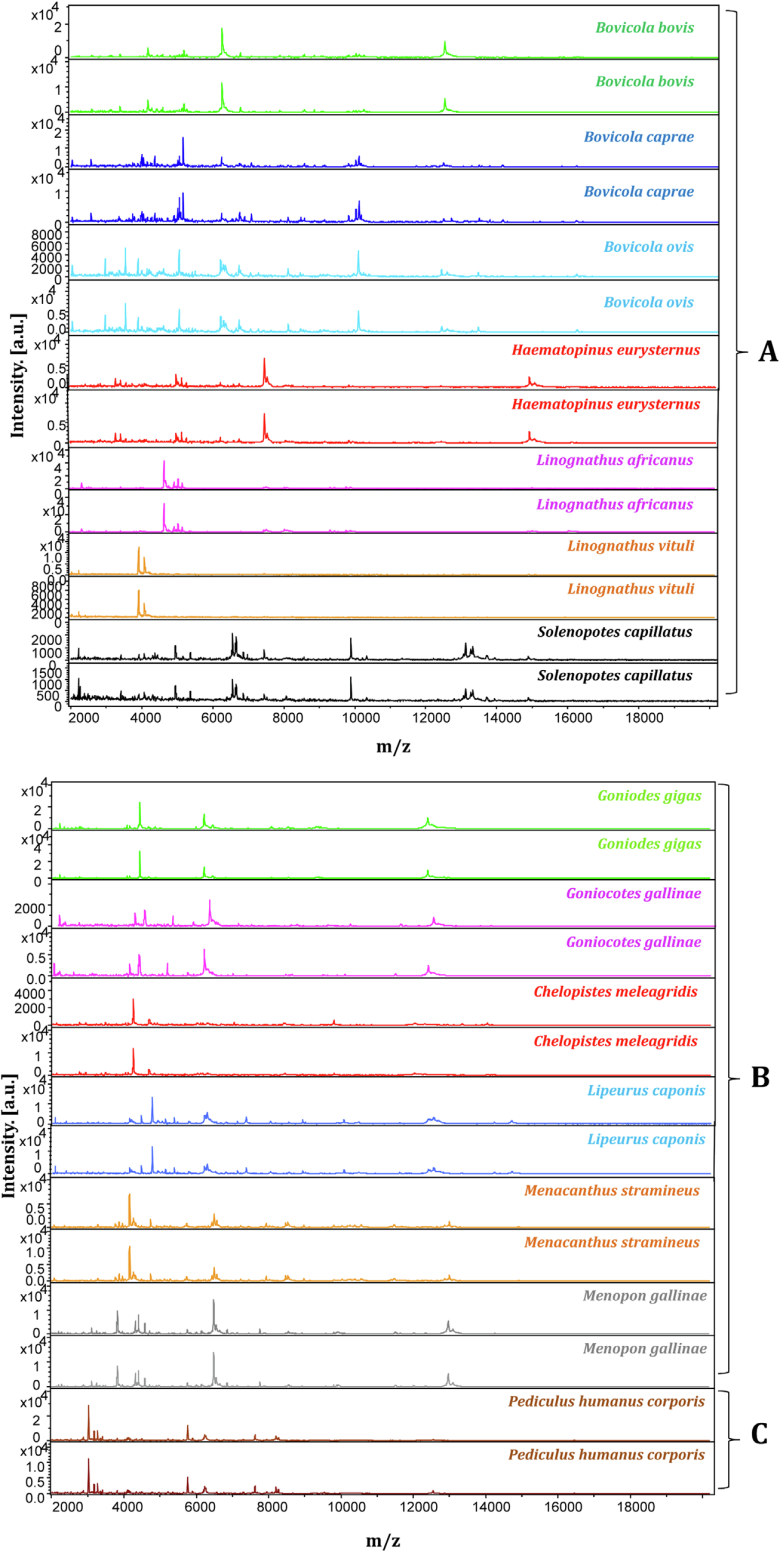
Good quality spectra of different species of mammalian and poultry lice visualized using FlexAnalysis software. (A: Mammal lice / B: Poultry lice / C: Human lice).

An analysis of the spectral profiles using FlexAnalysis software showed that the spectra obtained using the second protocol provided MALDI-TOF MS profiles of higher intensity and superior quality to those obtained with the first protocol (Fig. 4). Based on spectra quality MALDI-TOF MS, the second protocol provided good intra-species reproducibility and inter-species specificity between specimens of the same species and variability between different species. This protocol was therefore selected for further MALDI-TOF MS analyses (Fig. 5) to create a reference spectra database.

Therefore, protocol 2 was used for 408 specimens concerning the following species: *B. ovis*, *B. bovis*, *B*. *caprae*, *L. vituli*, *L*. *africanus*, *H. eurysternus*, *S*. *capillatus, C. meleagridis, Go. gigas, Me. stramineus, M. gallinae, G. gallinae, Li. caponis*, and *P. humanus corporis*. Samples were subjected to blind test analysis against the upgraded database (Table 4).

**Table 4:** MALDI-TOF MS identification of included louse species. Specimens were included based on the quality of their spectra (intensity, overall spectrum quality, and intra-species reproducibility).

Host	Species	Percentage of included specimens	Number of spectra added as reference	Number of specimens used for the blind test	LSVs obtained from blind tests against database & mean	Percentage of correct identification
Mammal lice	*Haematopinus eurysternus*	18/20 (90%)	4	14	[1.91–2.9] – 2.213	14/14 (100%)
	*Solenopotes capillatus*	59/68 (86.76%)	6	53	[1.719–2.707] – 2.023	52/53 (98.11%)
	*Linognathus vituli*	25/35 (71.42%)	5	20	[1.632–2.511] – 2.075	20/20 (100%)
	*Linognathus africanus*	17/21 (80.95%)	4	13	[1.704–2.644] – 2.046	13/13 (100%)
	*Bovicola caprae*	33/45 (73.33%)	8	25	[1.546–2.857] – 1.852	19/25 (76%)
	*Bovicola bovis*	29/40 (72.5%)	5	24	[1.728–2.873] – 2.11	23/24 (95.83%)
	*Bovicola ovis*	57/96 (59.37%)	9	48	[1.813–2.837] – 2.198	48/48 (100%)
Poultry lice	*Goniocotes gallinae*	5/5 (100%)	3	2	[2.34–2.393] – 2.366	2/2 (100%)
	*Goniodes gigas*	2/2 (100%))	1	1	1.932	1/1 (100%)
	*Menopon gallinae*	13/16 (81.25%)	4	9	[1.809–2.613] – 2.166	9/9 (100%)
	*Menacanthus stramineus*	6/10 (60%)	3	3	[2.306–2.608] – 2.456	3/3 (100%)
	*Chelopistes meleagridis*	15/18 (83.33%)	4	11	[1.703–2.046] – 1.824	11/11 (100%)
	*Lipeurus caponis*	2/8 (25%)	1	1	2.107	1/1 (100%)
Human lice	*Pediculus humanus corporis*	24/24 (100%)	0	24	[1.979–2.328] – 2.251	24/24 (100%)
	14	305/408 (74.75%)	57	248	2.115	240/248 (96.77%)

MALDI-TOF MS identification was considered correct when there was concordance between the morphological identification and molecular identification, when the latter was possible, that is when sequences were available in GenBank and were considered reliable.

In this study, we obtained 305 specimens with good quality spectra, of which 57 spectra were added as reference spectra and 248 specimens used for the blind test with an average LSV of 2.115 and correct identification percentages between 76% and 100% (Table 4). In all, 103 of the 408 samples (25.25%) tested had poor quality spectra and these were removed for this proof-of-concept (Supplementary Data 1). Nevertheless, specimens with low quality spectra were correctly identified with an average percentage of 61.11% and with low LSVs, highlighting the quality of the database created (Supplementary Data 1).

**Figure 6:**
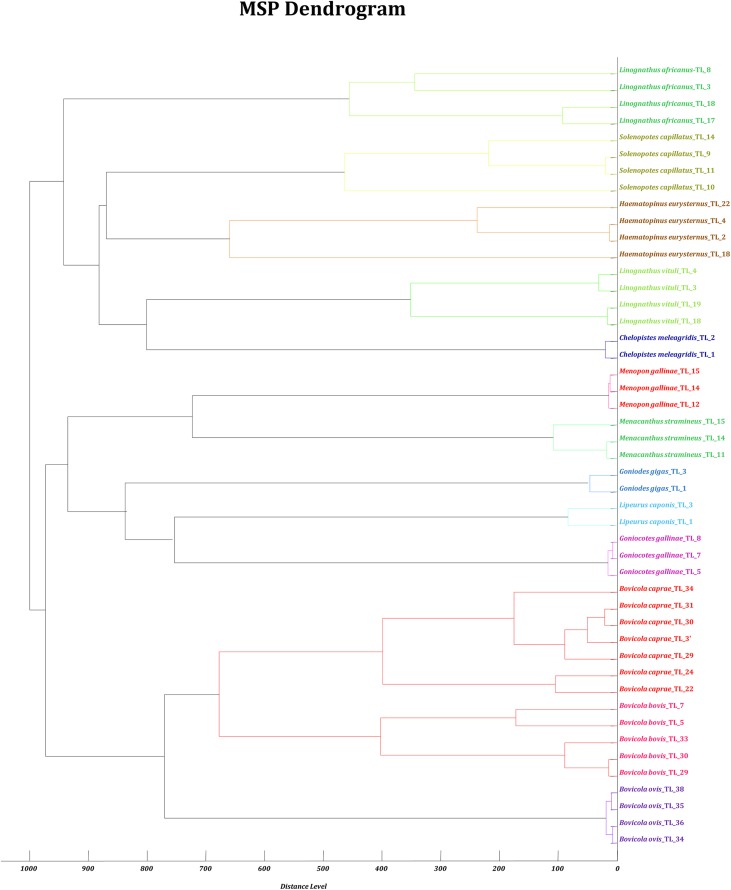
Dendrogram constructed using MALDI-Biotyper software v.3.3 including 2–7 random MS spectra representative of the 13 distinct species of lice.

The controls of fresh and non-engorged fresh *P. humanus corporis* lice were well identified at each test. The intra-species reproducibility and inter-species specificity of the MALDI-TOF MS profiles were further objectified using MALDI-Biotyper software cluster analysis. Dendrogram analysis revealed specific clustering on distinct branches of lice according to species. Lice belonging to the same genus were grouped in the same part of the MSP dendrogram (Fig. 6).

## Discussion and conclusion

The morphological identification of lice is very complex because the species are morphologically close to one another. For the first time, MALDI-TOF MS was used as an additional tool for lice identification.

In this study, we successfully identified 14 species of lice using MALDI-TOF MS. Morphological identification was molecularly confirmed by targeting a fragment of louse *18S* rRNA gene sequences. The choice of the *18S* rRNA gene is based on previous results that proved the relevance of this gene for louse identification and the presence of reference sequences in GenBank [21]. However, 5/13 of the lice species studied in this work had no sequence available in GenBank, highlighting the drawbacks of using molecular biology alone for louse identification. Only 4/13 species of lice presented correct identification using the *18S* rRNA gene. The remaining 4/13 species of lice resulted in incorrect identification despite the fact that their reference sequences were present in GenBank. Further analysis of the GenBank reference sequences of each of these species revealed that they were all of poor quality.

This study allowed us to add five new sequences that did not exist on GenBank, and eight additional complementary sequences for which a reference was already available (Table 3). Five of the 13 sequences of lice namely *Go. gigas, B. bovis, B. ovis, B. caprae,* and *Chelopistes meleagridis* were already published under new genera (Table 5) [14, 34, 38, 42, 47].

**Table 5:** Hosts and distribution of lice in the orders Mallophaga and Anoplura.

Order/suborder/family	Valid name	References	Host
Mallophaga, Ischnocera, Philopteridae	*Goniodes gigas* (Taschenberg, 1879) (=*Goniocotes gigas* Taschenberg, 1879)	[34]	Poultry
Mallophaga, Ischnocera, Trichodectidae	*Bovicola bovis* (Linnaeus, 1758) (= *Damalinia bovis* Linnaeus, 1758)	[14]	Cattle
Mallophaga, Ischnocera, Trichodectidae	*Bovicola ovis* (Schrank, 1781) (=*Damalinia ovis* (Schrank, 1781)	[14]	Sheep
Mallophaga, Ischnocera, Trichodectidae	*Bovicola caprae* (Gurlt, 1843) (=*Damalinia caprae* (Gurlt, 1843)	[14]	Goat
Mallophaga, Ischnocera, Philopteridae	*Chelopistes meleagridis* (Linnaeus, 1758) (= *Goniodes meleagridis* (Linnaeus, 1758)	[14, 34]	Poultry
Mallophaga, Amblycera, Menoponidae	*Menacanthus stramineus* Nitzsch, 1818	[42]	Poultry
Mallophaga, Ischnocera, Philopteridae	*Lipeurus caponis* Linnaeus, 1758	[42]	Poultry
Mallophaga, Amblycera, Menoponidae	*Menopon gallinae* Linnaeus, 1758	[42]	Poultry
Mallophaga, Ischnocera, Philopteridae	*Goniocotes gallinae* de Geer, 1778	[38]	Poultry
Anoplura, Haematopinidae	*Haematopinus eurysternus* Nitzsch, 1818	[42, 47]	Cattle
Anoplura, Linognathidae	*Solenopotes capillatus* Enderlein*,* 1904	[42]	Cattle
Anoplura, Linognathidae	*Linognathus vituli* Linnaeus, 1758	[42]	Cattle
Anoplura, Linognathidae	*Linognathus africanus* Kellogg & Paine, 1911	[47]	Goat

A preliminary MALDI-TOF MS database containing the spectra of 14 species was hereby created and the database will be regularly updated with the spectra of new specimens. The spectra files are available on request and transferable to any Bruker MALDI-TOF MS device. The MALDI-TOF MS arthropod database can be shared through scientific collaboration projects; it will be possible to freely query this database online in the future.

For use in entomology, the choice of arthropod body parts to be used for the MALDI-TOF MS test is a very important criterion. For ticks and mosquitoes, MALDI-TOF MS identification of the arthropod species is based on leg spectra. Other body parts had to be carefully selected for other arthropods when the legs did not provide satisfactory spectra [10, 13, 22]. Here, the spectral profiles generated from the cephalothorax-legs of the lice subjected to MALDI-TOF MS were reproducible. Spectral analysis highlighted intra-species reproducibility and inter-species specificity, which was consistent with the morphological classification. In addition, hierarchical clustering based on the MALDI-TOF MS spectra revealed that all of the specimens from the same species were grouped in the same branch. Our results demonstrated that the use of the body of a louse without the abdomen was the best sample for distinguishing lice species using the MALDI-TOF MS approach. There are many advantages to selecting this part of the body, for example avoiding the influence of the intestinal contents on the MALDI-TOF MS spectra [50]. Moreover, using a small body part for MALDI-TOF MS allows further analyses of the remaining parts of the arthropod, such as the detection of microorganisms [10] or the identification of blood meals of the arthropods [31]. Higher quality spectra resulted from the cephalothorax-legs part of the louse compared to when it was dissected longitudinally (Fig. 4). This can be explained by the fact that some parts of arthropods yield better spectral qualities than others, as has been demonstrated by several studies [10, 13, 22, 43].

In this study, we included only good quality spectra. Indeed, at this stage, only high-quality spectra can be included to validate the results and create a reliable database.

The number of specimens with low quality spectra can be explained by the fact that all these samples had to be frozen and thawed several times for various analyses including long morphological identification, molecular biology, and MALDI-TOF MS assays. These repeated thawing steps could have caused protein alterations responsible for the poor quality of the spectra. This hypothesis is supported by the fact that the groups of samples that were manipulated first have a greater number of high-quality spectra. This should not be an issue when applied to entomological studies since molecular biology is not always required and when a comprehensive MALDI-TOF MS database is available, the quality of the spectra will be improved. Nevertheless, many specimens with low quality spectra were correctly identified, reaching 100% correct identification for some species such as *Menopon gallinae* (average: 61.11%) (Supplementary Data 1). The performance of the identification despite the many thawing steps is a validation of the quality of the database created, which will be continuously strengthened with new field specimens.

MALDI-TOF MS enables the identification of lice without any entomological knowledge [25], as long as the database is comprehensive. Furthermore, the MALDI-TOF MS sample preparation method is simple and the speed of data analysis makes it possible to obtain quick and reliable identification results [50].

This study points to new possibilities for improving the knowledge of animal lice in Algeria by using several identification tools. We have also illustrated the limitations of molecular biology with the lack of comprehensiveness of the NCBI GenBank database, which is a major setback to using this method. To circumvent these limitations, we have deposited our new sequences in the NCBI GenBank database (Table 3).

This fast and accurate low-cost tool identifies not only the different immature stages of the arthropod’s life cycle [6, 13], but also the origin of blood meal sources from arthropods [31].

Recently, preliminary studies have examined the ability of MALDI-TOF MS to detect *Plasmodium* parasites in *Anopheles* mosquitoes [23], differentiate ticks infected or not infected with *Borrelia* spp. or spotted fever group *Rickettsia* spp. [3, 10, 11, 43, 49], and fleas infected or not with *Bartonella* spp. [10].

The MALDI-TOF MS detection of louse-borne bacteria could provide new opportunities for vector surveillance, particularly in Algeria where all these louse species are present [16].

Previous studies reported the detection of *Rickettsia slovaca* in *Haematopinus suis* from Algeria [51]. It was later demonstrated that lice could acquire the bacterium *R. slovaca* after feeding on a bacteremic boar which does not yet prove that they are vectors, but would require epidemiological studies to be carried out [51]. It would be interesting to attempt to detect louse-associated bacteria such as *Bartonella quintana* or *Borrelia recurrentis*. Using the proposed protocol, the abdomen of the lice can be used for molecular screening of microorganisms.

This study confirmed that MALDI-TOF MS is a faster and cheaper method for identifying lice stored at −20 °C. In the field, alcohol is a more widely-used method of conserving the samples, especially in countries with limited resources [5]. It has been shown that MALDI-TOF MS is reliable for identifying arthropods preserved in alcohol, such as ticks[5], mosquitoes [29], and fleas [10]. Therefore, it would be interesting in the future to set up a MALDI-TOF MS protocol for identifying lice kept in alcohol [52]. It would also be interesting to assess whether MALDI-TOF MS can be used to differentiate lice which are infected or not infected by louse-borne microorganisms.

Supplementary materials

Supplementary material is available at https://www.parasite-journal.org/10.1051/parasite/2020026/olm

*Supplementary Data 1*. MALDI-TOF MS identification of louse species excluded from the final analyses.
