# Supplementary material for: Development of MALDI-TOF mass spectrometry for the identification of lice isolated from farm animals
Source: Parasite. 2020 Apr 30;27:28. doi: 10.1051/parasite/2020026 (PMC7191974; doi:10.1051/parasite/2020026)
Supplement: Supplementary file 1 [file parasite-27-28-s1.pdf]

**Supplementary data 1**. MALDI-TOF MS identification of louse species excluded from the final analyses.

| Host | Species | Percentage of excluded specimens | LSVs obtained from blind tests against Database | Percentage of correct identification |
|---|---|---|---|---|
| Mammal lice | *Haematopinus eurysternus* | 2 (10%) | [1.687-1.878] -1.782 | 1/2 (50%) |
| | *Solenopotes Capillatus* | 9 (13.23%) | [1.304-1.698] -1.561 | 7/9 (77.77%) |
| | *Linognathus vituli* | 10 (28.57) | [1.198-1.781] 1.596 | 9/10 (90%) |
| | *Linognathus africanus* | 4 (19.05%) | [1.242-1.613] -1.432 | 3/4 (75%) |
| | *Bovicola caprae* | 12 (26.66) | [1.269-2.031] | 8/12 (66.66 |

| | | | | |
|---|---|---|---|---|
| | | | 1.573 | %) |
| | *Bovicola bovis* | 11 (27.5%) | [1.321-2.729] 1.811 | 3/11(27.27%) |
| | *Bovicola ovis* | 39 (40.62%) | [1.188-2.658] 1.403 | 21/39 (53.84%) |
| Poultry lice | *Goniocotes gallinae* | / | / | / |
| | *Goniodes gigas* | / | / | / |
| | *Menopon gallinae* | 3 (18.75%) | [1.605-1.803] -1.691 | 3/3 (100%) |
| | *Menacanthus stramineus* | 4 (40%) | [1.075-1.439] 1.276 | 0% |
| | *Chelopistes meleagridis* | 3/18 (37.5%) | [1.536-1.627] -1.594 | 0% |
| | *Lipeurus caponis* | 6 (75%) | / | / |
| Human lice | 13 | 103 (25.25%) | 1.572 | 55/90 (61.11 |

%)
